# Side effects of oxytocin and carbetocin for mangement at third trimester

**DOI:** 10.6026/9732063002001405

**Published:** 2024-10-31

**Authors:** Akshay Anup Bahekar, Rajkumar P Patange, Yamini Patil

**Affiliations:** 1 Department of Obstetrics and Gynaecology, Krishna Institute of Medical Sciences, Karad - 415110, Maharashtra, India

**Keywords:** Third stage of labour, vitals, side effects, blood loss, delivered, carbetocin, oxytocin

## Abstract

The third stage of labor lasts from the time the baby is born until the maternal placenta is delivered. Therefore, it is of interest to evaluate the effectiveness and side effects of oxytocin and carbetocin for third-trimester labor management. Hence, we divided 95 patients into two groups: group I received oxytocin and group II received carbetocin. We measured blood loss at 72 hours duration and further examined vitals as well as side effects after delivery. We found that there was no significant difference in side effects between the two groups (p value = 0.8). However, that carbetocin appeared to be a more effective alternative for minimizing blood loss in the third stage of labor.

## Background:

Due to the risk of postpartum hemorrhage (PPH), the 3^rd^ stage of labour is regarded as being the most critical part of childbirth [1]. Primary postpartum hemorrhage (PPH) is characterized by the expulsion of more than 500 ml of blood from the genital tract within 24 hours following vaginal birth, according to the World Health Organization's definition [2]. Three basic procedures make up active labour management: giving the infant an uterotonic medicine after birth, clamping and cutting the umbilical cord early, and applying regulated tract pressure on the chord while waiting for the placenta to separate and the baby to be born [3]. While standard uterotonic like oxytocin have been utilized to prevent PPH, they possess certain limitations, such as a shorter half-life, a reduced contraction length, and a greater incidence of side effects such as fluid overload, convulsions, arrhythmia, and pulmonary edema [4]. However, the cold chain required for preserving oxytocin is not sufficiently maintained in many places, including our own nation, increasing the risk of treatment failure [5]. Therefore, in order to successfully prevent bleeding caused by uterine atony, it is crucial to use a powerful uterotonic medication. Although Oxytocin has traditionally been used for this purpose, its effectiveness and stability problems have led to the exploration of alternative options. Carbetocin, a durable artificial derivative of Oxytocin with comparable clinical and pharmacological characteristics, presents itself as a hopeful substitute. Six the extended duration of its activity (about 1 hour) results in longer contraction time and less negative effects in comparison to Oxytocin. Carbetocin remains effective for duration of 16 hours after a solitary administration, hence decreasing the likelihood of postpartum hemorrhage during vaginal birth. In addition, it provides enhanced contractions and reduced side effects such as headache, tremors, low pressure of blood, nausea, stomach discomfort, and itching. Existing literature indicates that administering Carbetocin as a preventive measure might be a suitable substitute for Oxytocin in the prevention of PPH [6]. Therefore, it is of interest to report the side effects of oxytocin and carbetocin in active management of 3^rd^ stage of labour among Indian women.

## Materials and Methods:

The current hospital based observational prospective study was conducted after admission into ward no 12 for vaginal delivery for over a period of one and half years with 95 patients in total in the department of obstetrics and gynecology. The eligible patients were randomly divided into 2 equal groups *i.e.* Oxytocin (Group I, 10 IU intramuscular) and Carbetocin (Group II 100 microgram Carbetocin diluted in 10 ml of normal saline injected intravenously over 1-2 minutes). Blood loss during the 3^rd^ stage of labour was quantified using a BRASSS-V DRAPE blood-collecting bag. Over 500 milliliters loss was managed by oxytocics. Furthermore, loss and duration were recorded with stopwatch. Maternal hemoglobin and hematocrit levels were reassessed 72 hours after delivery. Moreover, post-delivery patients were further monitored around 2 hours for vital signs, vaginal bleeding, any side effects, including nausea, vomiting, shivering, fever and diarrhea. Additional urotonics are methyl ergometrine, misoprostol & carboprost.

## Inclusion criteria:

All patients undergoing full term vaginal delivery.

## Exclusion criteria:

[1] Bleeding disorders and or coagulation defect.

[2] Those under anticoagulant therapy.

[3] Placenta previa, placenta accretes.

[4] Preeclampsia or HELLP syndrome.

[5] Renal and heart diseases.

[6] Hypersensitivity to drugs.

[7] Multiple pregnancy.

## Statistical analysis:

The SPSS program, version 16, was used for the computerization and analysis of the data. The chi-square test was used for the purpose of comparing qualitative variables, whilst the t-test was utilized for the purpose of comparing quantitative data. When a p-value was less than 0.05 and a confidence range of 95% was used, statistical significance was determined as being present.

## Results:

Table 1 shows that, in Group I, which received Oxytocin, 70 women were aged 18 to 25, 21 were aged 26 to 30, and 4 were over 30 years old. In Group II, which received low-dose Carbetocin, 67 women were aged 18 to 25, 22 were aged 26 to 30, and 6 were over 30 years old. The mean ages were 24 and 24.3 years for Groups I and II, respectively, with standard deviations of 3.7 and 3.8. The p-value of 0.5 indicates that there was no statistically significant difference in age distribution between the two groups, suggesting that age was evenly matched across the study. Table 2 shows that, group I had 44 primiparous (first-time mothers), 43 multiparous (having given birth more than once), and 8 grand multiparous women (having given birth five or more times). Group II included 42 primiparous, 42 multiparous and 11 grand multiparous women. The p- value of 0.76 suggests there was no significant difference in parity between the groups, indicating that both groups had a similar distribution of women based on the number of previous births. Table 3 shows that, group I had 86 women who were booked (registered for prenatal care) and 9 who were unbooked. In Group II, 83 women were booked and 12 were unbooked. The p-value of 0.2 indicates no significant difference in registration status between the two groups, showing that the proportion of women who received prenatal care was comparable between the groups. Table 4 shows that, in group I, 39 women had labour augmented (labour that was assisted or stimulated), 10 had labour induced (labour that was medically initiated), and 46 had spontaneous labour (natural onset of labour). Group II had 39 women with augmented labour, 8 with induced labour, and 48 with spontaneous labour. The p-value of 0.87 suggests no significant difference in the type of labour between the groups, indicating similar labour patterns in both groups.

Table 5 shows that, group I at 38 weeks and group II at 39 weeks. The standard deviations were 1.1 and 1 week, respectively. The p-value of 0.5 indicates no significant difference in gestational age at delivery between the groups, showing that the timing of birth was consistent across the study population. Table 6 shows that, the mean hemoglobin (Hb) levels before delivery were 9.8 g/dL in group I and 9.7 g/dL in group II, with standard deviations of 1.3 and 1.5, respectively. After delivery, the mean Hb levels were 9.1 g/dL in group I and 9.2 g/dL in group II, with standard deviations of 1.4 and 1.4. The p-value for Hb before delivery was 0.6, indicating no significant difference, while the p-value for Hb after delivery was 0.04, indicating a significant difference. The duration of the third stage of labour averaged 6.6 minutes in group I and 6.2 minutes in group II with a p-value of 0.1 whereby showing no significant difference in the duration. Table 7 shows that, 14 women in Group I required additional uterotonics, compared to only 3 women in group II. The majority in both groups did not need additional uterotonics (81 in group I and 92 in group II). The p-value of 0.002 indicates a significant difference, with group I having a higher need for additional uterotonics, suggesting that low-dose Carbetocin might be more effective in reducing the need for additional uterotonics compared to Oxytocin. Table 8 shows that, PPH occurred in 3 women in group I and 1 woman in group II. The majority of women did not experience PPH (92 in Group I and 94 in Group II). The p-value of 0.15 indicates no significant difference in the incidence of PPH between the groups, although numerically fewer cases were observed in the Carbetocin group. Table 9 shows that, in group i, each of the three causes atonic, polyhydramnios, and grand multipara - contributes equally at 33.3%. In group ii, polyhydramnios is the sole cause, representing 100% of the cases. The total percentages for each group add up to 100%, reflecting the distribution of causes within the respective groups. Table 10 shows that, the majority in both groups did not require transfusions (92 in Group I and 94 in Group II). The p-value of 0.15 indicates no significant difference in the need for blood transfusions between the groups, suggesting similar clinical management outcomes in terms of transfusion requirements. Table 11 shows that, all women in both groups survived, with no maternal deaths reported in either group. The p-value of 1 indicates no significant difference in maternal outcomes between the groups, confirming that both treatments were equally safe with respect to maternal survival. Table 12 shows that, in group I, side effects include Nausea (2.1%), Vomiting (1.1%), abdominal pain (1.1%), Headache (2.1%), Hypotension (1.1%), Arrhythmia (2.1%), and Fever (1.1%). In group II, the side effects include Nausea (1.1%), abdominal pain (1.1%), Headache (1.1%), and Fever (1.1%). The p-value of 0.8 indicates that there is no statistically significant difference (NS) between the two groups in terms of the side effects experienced.

## Discussion:

Various studies have shown that, many drugs and methods of administration have been extensively researched to assess their effectiveness in managing the third stage of labor. However, significant variations persist in the approaches used worldwide. During childbirth, oxytocin is commonly used as a pharmaceutical agent to effectively prevent and manage excessive hemorrhaging. Its use is endorsed by the World Health Organization (WHO) because of its rapid effectiveness and low likelihood of causing high blood pressure or muscle spasms. However, the effectiveness of Oxytocin may be limited by the dosage, as the saturation of Oxytocin receptors in the myometrium can reduce its potency. Higher doses can lead to negative effects such as narrowing of the coronary arteries, decreased blood pressure, and excessive water retention due to the antidiuretic properties. While Oxytocin is frequently utilized, it may not always be the most potent option and may require the addition of other medications to effectively manage blood loss [7, 8-9]. The present study shows that the age distribution was similar between the Oxytocin and low-dose Carbetocin groups. In both groups, the majority of women fell within the 18-25 years and 26-30 years age brackets. The mean ages were 22.78 ± 2.82 years in the Oxytocin group and 22.50 ± 2.8 years in the Carbetocin group, with a non-significant difference (Z = 0.067, p > 0.05). This suggests that age was evenly matched across both treatment groups, indicating comparable baseline characteristics. In addition to above, we also found, no significant difference was observed in parity distribution between the Oxytocin and Carbetocin groups. Both groups had similar proportions of primiparous, multiparous, and grand multiparous women. This parity distribution ensures that the study groups were balanced in terms of previous childbirth experiences, which is crucial for comparing treatment outcomes effectively.

Study by Algharib *et al.* showed that mean age in Group a (Carbetocin), the age ranged from 27 to 43 years, with a mean ± standard deviation of 34.85 ± 5.034 years. In Group B (Oxytocin), the age ranged from 26 to 41 years, with a mean ± standard deviation of 33.71 ± 4.522 years [10]. Study by Larciprete *et al.* showed that mean age of Group O was 36.1 and group C was 37.1 years (p0.33) [11] and in study by Jannu *et al.* showed that mean age of Group O was 26.8 and group C was 26.1 years (p0.06) [12]. Study by Fahmy *et al.* showed that mean age of Group O was 24.5 and group C was 25.4 years (p0.24) [13]. Study by Fahmy *et al.* showed that mean parity for group O was 4 and C was 3 [13]. Our study found that, the registration status for prenatal care was comparable between the Oxytocin and Carbetocin groups. The majority of women in both groups were booked for prenatal care, with no significant difference observed (p = 0.2). This similarity in registration status indicates uniformity in access to antenatal services and baseline monitoring among participants. Moreover, both treatment groups exhibited similar distributions in the type of labor experienced. Augmented, induced, and spontaneous labor were evenly distributed between the Oxytocin and Carbetocin groups, with no significant disparity (p = 0.87). This balance in labour types ensures that any observed differences in outcomes can be attributed to the treatment rather than variations in labour induction or management. Gestational age at delivery was closely matched between the Oxytocin and Carbetocin groups. The mean gestational ages were 38weeks and 39 weeks, respectively, with no statistically significant difference observed (p = 0.2). This similarity suggests that both groups were representative of pregnancies at term, minimizing confounding effects related to gestational age. Hemoglobin levels before delivery did not differ significantly between the Oxytocin and Carbetocin groups, with similar mean values (9.8 g/dL vs. 9.7 g/dL) and standard deviations (p = 0.6). However, hemoglobin levels after delivery showed a significant difference (p = 0.04), indicating lower postpartum hemoglobin levels in the Carbetocin group. Study by Larciprete *et al.* 47 showed that mean Hb of Carbetocin group was 11.4 and Oxytocin group was 11.8 g/dL and post-delivery it decreased by 0.7 in Carbetocin group and 1.1 in Oxytocin group. Study by Fahmy *et al.* 60 showed that mean HB of O group was 9.8 and C was 10 g/dL. The duration of the third stage of labour averaged 6.6 minutes in Group I and 6.2 minutes in Group II, the p-value of 0.1 showing no significant difference in the duration. The duration of the third stage of labour was similar between groups (p = 0.1), suggesting consistent management of labour duration. In addition to above, a significantly fewer women in the Carbetocin group required additional uterotonic compared to the Oxytocin group (p = 0.002). This finding underscores the effectiveness of low-dose Carbetocin in reducing the need for supplementary uterotonic, potentially minimizing complications associated with excessive bleeding postpartum. Research conducted by Algharib *et al.* revealed that Group A (Carbetocin) had a mean gestational age of 37- 40 weeks with a standard deviation of 38.54±1.176 weeks, while Group (B) had a mean gestational age ranging from 37-40 weeks with a standard deviation of 38.59±1.111 weeks. There were no significant differences between the groups, as shown by the P-value of 0.754 [10]. Another study showed that mean gestational age among Carbetocin group was 38 and Oxytocin group was 37 weeks (p=0.01). Similar findings were seen in present study [11]. In the study by Abdeen *et al.* the mean ± S.D. gestational age in Group A (Carbetocin) was 38.61 ± 1.11 weeks, whereas in Group B (those who received a combination of intravenous Oxytocin and intramuscular ergometrine), it was 38.31 ± 1.11 weeks [14]. A previous study conducted by Moertl *et al.* demonstrated that patients treated with Oxytocin experienced more significant hypotension and hemodynamic rebound compared to those who received Carbetocin [15]. Compared to oxytocin, carbetocin may have a clinically relevant effect on reducing nausea and vomiting. However, this difference did not reach statistical significance because the study was not powerful enough. Both medications exhibit comparable effects on the management of nausea and vomiting. Both products exert comparable effects on blood pressure, heart rate, the requirement for vasopressors, and blood loss [16]. It is possible that carbetocin could be used instead of oxytocin to help stop postpartum hemorrhage (PPH) in the third stage of labor in women who are having induced or augmented labor. This would lessen the necessity of manually removing the placenta. Midwives are able to have their hands free to concentrate on other procedures that are more necessary following the birth of the fetus when they administer carbetocin by intravenous infusion. This is especially helpful in busy clinical practices [17].

## Conclusion:

Group I experienced more side effects, such as nausea and vomiting, compared to group II. The incidence of postpartum hemorrhage, the need for blood transfusion and maternal outcomes did not significantly differ between the groups, highlighting the overall safety of both drugs. However, the higher rate of side effects in the oxytocin group suggests a need for careful monitoring and management of these adverse effects.

## Figures and Tables

**Table 1:** Age distribution

**Age in years**	**Group I**	**%**	**Group II**	**%**
18 to 25	70	74	67	71
26 to 30	21	22	22	23
> 30	4	4.2	6	6.3
Total	95	100	95	100
Mean	24		24.3	
SD	3.7		3.8	
P value	0.5		NS	

**Table 2:** Parity distribution

**Parity**	**Group I**	**%**	**Group II**	**%**
Primi para	44	46.3	42	44.2
Multi para	43	45.3	42	44.2
Grand multipara	8	8.4	11	11.6
Total	95	100	95	100
P value	0.76		NS	

**Table 3:** Distribution on registration

**Registered**	**Group I**	**%**	**Group II**	**%**
Booked	86	90.5	83	87.4
Un booked	9	9.5	12	12.6
Total	95	100	95	100
P value	0.2		NS	

**Table 4:** Type of labour

**Type**	**Group I**	**%**	**Group II**	**%**
Augmented	39	41.1	39	41.1
Induced	10	10.5	8	8.4
Spontaneous	46	48.4	48	50.5
Total	95	100	95	100
P value	0.87		NS	

**Table 5:** GA

**Gestational age in Weeks (GA)**	**Group I**	**Group II**
Mean	38	39
SD	1.1	1
P value	0.5	NS

**Table 6:** Laboratory finding

**Parameters**	**Group I**		**Group II**		**P value**
	**Mean**	**SD**	**Mean**	**SD**	
Hb before delivery	9.8	1.3	9.7	1.5	0.6
Hb after delivery	9.1	1.4	9.2	1.4	0.04*
Duration of third stage of labour	6.6	2.3	6.2	1.6	0.1

**Table 7:** Uterotonic use

**Additional use**	**Group I**	**%**	**Group II**	**%**
Yes	14	14.7	3	3.2
No	81	85.3	92	96.8
Total	95	100	95	100
P value	0.002		S	

**Table 8:** PPH Distribution

**PPH**	**Group I**	**%**	**Group II**	**%**
Yes	3	3.2	1	1.1
No	92	96.8	94	98.9
Total	95	100	95	100
P value	0.15		NS	

**Table 9:** pph distribution

**Cause**	**Group I (n=3)**	**%**	**Group ii (n=1)**	**%**
Atonic	1	33	0	0
Polyhydramnios	1	33	1	100
Grand multipara	1	33	0	0
Total	3	100	1	100
P value	0.51 NS			

**Table 10:** Blood transfusion distribution

**Blood transfusion**	**Group I**	**%**	**Group II**	**%**
Required	3	3.2	1	1.1
Not required	92	96.8	94	98.9
Total	95	100	95	100
P value	0.15		NS	

**Table 11:** Outcome

**Outcome**	**Group I**	**%**	**Group II**	**%**
Survived	95	100	95	100
Died	0	0	0	0
Total	95	100	95	100
P value	1		NS	

**Table 12:** Side effects

**Side effects**	**Group I**	**%**	**Group II**	**%**
Nausea	2	2.1	1	1.1
Vomiting	1	1.1	0	0
Abdominal pain	1	1.1	1	1.1
Headache	2	2.1	1	1.1
Hypotension	1	1.1	0	0
Arrhythmia	2	2.1	0	0
Fever	1	1.1	1	1.1
P value	0.8 NS			

## References

[1] Akhter P (2018). Mymensingh Medical Journal: MMJ..

[2] Gulmezoglu AM (2009). WHO Guidelines Approved by the Guidelines Review Committee..

[3] Prendiville WJ (2000). Cochrane database of systematic reviews..

[4] Ashraf F (2021). Bioresearch Communications..

[5] Kane TT (1992). Studies in family planning..

[6] Attilakos G (2010). BJOG: An International Journal of Obstetrics & Gynaecology..

[7] Vaid A (2009). Archives of gynecology and obstetrics..

[8] Pk D (1988). Acta Obstetricia et Gynecologica Scandinavica..

[9] Patil AS (2016). Al Ameen Journal of Medical Sciences..

[10] Algharib MA (2021). Al-Azhar International Medical Journal..

[11] Larciprete G (2013). J Prenat Med..

[12] Jannu V (2023). Journal of Obstetric Anaesthesia and Critical Care..

[13] Fahmy NG (2016). Egyptian Journal of Anaesthesia..

[14] Abdeen EZE, Shehata NA (2018). Evidence Based Women's Health Journal..

[15] Moertl MG (2011). An International Journal of Obstetrics & Gynaecology..

[16] Mannaerts D (2018). Journal of pregnancy..

[17] Liu H (2020). Maternal-Fetal Medicine..

